# Sugar-Sweetened Coffee Intake and Blood Glucose Management in Korean Patients with Diabetes Mellitus

**DOI:** 10.3390/metabo12121177

**Published:** 2022-11-25

**Authors:** Hyeonji Yoo, Kyong Park

**Affiliations:** Department of Food & Nutrition, Yeungnam University, 280 Daehak-ro, Gyeongsan 38541, Republic of Korea

**Keywords:** diabetes mellitus, coffee, glycemic control, diabetes management, Korean

## Abstract

One of the most frequently consumed beverages by Korean adults is 3-in-1 coffee (mixed coffee) with sugar and creamer. Hence, understanding the effect of sugar-sweetened coffee (SSC) consumption on blood sugar levels in patients with diabetes mellitus (DM) is important. This study was conducted using the Korea National Health and Nutrition Examination Survey data from 2008 to 2020. In total, 5671 patients with DM were included in the analysis. Coffee consumption patterns were assessed using a 24 h recall. Fasting blood glucose (FBG) and hemoglobin A1c (HbA1c) levels were used to evaluate whether patients reached the glycemic control targets defined by the Korean Diabetes Association. In total, 46.57% of patients with DM included in this analysis consumed SSC. Patients who frequently consumed SSC had significantly higher FBG and HbA1c levels than those who did not (both *p* for trend <0.01). In a multivariate logistic regression model, the odds ratios of not achieving the target FBG and HbA1c levels were 1.24 (95% confidence interval [CI]: 1.03–1.48, *p* for trend = 0.01) and 1.29 (95% CI: 1.05–1.58, *p* for trend = 0.009), respectively. Frequent consumption of SSC can cause difficulty in FBG and HbA1c management in patients with DM.

## 1. Introduction

The global prevalence of diabetes mellitus (DM) has been increasing; the effects have been seen in Korea as well [1]. The prevalence of DM was approximately 10.7% among Korean adults in 2020, which was the highest rate of prevalence in the past decade [2]. According to the 2020 Causes of Death Statistics in Korea, the cause-specific death rate of DM was 16.5 per 100,000 population, which makes it one of the biggest national health concern [3]. Glycemic control is crucial for reducing the DM-related mortality rate through the prevention and/or delay of diabetes complications, such as renal failure or cardiovascular disorders. Excessive intake of added sugars can worsen glycemic control; sugar-sweetened beverages (SSBs) are a main source of added sugars in the Korean diet [4,5]. Consumption of SSBs increased by approximately two-fold between 1998 and 2009 in Korea [6], and coffee ranks high among the most consumed SSBs by Korean adults. According to the 2020 National Food & Nutrition Statistics, coffee ranked second among the most popular food among Koreans [7]; it moved up one place in the rankings in 2019, before which it was ranked third behind cabbage kimchi and rice [8]. In Korea, 150,780 tons of coffee was consumed in 2020–2021, with a steady increase of 2.0% in the annual coffee consumption over the past 4 years [9]. However, Koreans mainly consume sugar-sweetened coffee (SSC), including 3-in-1 coffee (mixed coffee) with sugar and creamer; approximately 73.7% men and 63.0% women consume SSC or 3-in-1 coffee in Korea [10]. This trend is more prominent among middle-aged adults who have an increased risk of metabolic syndrome. 

Previous epidemiological studies have consistently reported that coffee can lower the risk of DM. The US Nurses’ Health Study and the Health Professionals Follow-up Study have reported that participants who increased their daily coffee consumption showed a 11% lower risk of DM, whereas those who decreased their daily coffee consumption showed a 17% higher risk of DM [11]. Furthermore, in a meta-analysis on the association between coffee consumption and the risk of DM among Asians, the occurrence of DM was significantly lower in the group that consumed more coffee than in the group that consumed less coffee [12]. However, most studies did not differentiate between sugarless black coffee and SSC. Therefore, studies on the effects of coffee additives are essential, especially for Koreans who prefer 3-in-1 coffee. In addition, investigation of the effects of SSC consumption on glycemic control among people with DM is important and critical. Thus, this study aimed to examine the association between SSC intake levels and glycemic control and the achievement of the glycemic target among Korean patients with DM.

## 2. Methods

### 2.1. Study Population

The Korea National Health and Nutrition Examination Survey (KNHANES) is an ongoing annual national cross-sectional survey prescribed by Article 16 of the National Health Promotion Act [13]. The KNHANES generates statistics on health status and behaviors and food and nutrition intake among Koreans; these statistics are used as basic data for health policies, such as development of health promotion programs and establishment and assessment of the National Health Plan [14].

This study analyzed KNHANES data from 2008 to 2020. In total, 108,497 individuals responded to the survey; only patients with confirmed DM were considered eligible. DM was identified based on a self-reported medical history of DM (e.g., physician’s diagnosis) or medical treatment with insulin or oral hypoglycemic medication.

Among the 6337 patients with DM aged ≥30 years in the surveys conducted between 2008 and 2020 (excluding cases with missing sampling weight data), 2 women who were pregnant or lactating at the time of the survey, 575 individuals with missing data on fasting blood glucose (FBG) or hemoglobin A1c (HbA1c) levels, and 89 individuals with invalid total daily energy intake values (<500 kcal or >5000 kcal) were excluded. Finally, 5671 individuals were included in the analysis.

The KNHANES was conducted with approval from the Institutional Review Board (IRB) of the Korea Disease Control and Prevention Agency (KDCA)—Approval Nos: 2008-04EXP-01-C, 2009-01CON-03-2C, 2010-02CON-21-C, 2011-02CON-06-C, 2012-01EXP-01-2C, 2013-07CON-03-4C, 2013-12EXP-03-5C, 2018-01-03-P-A, 2018-01-03-C-A, and 2018-01-03-2C-A. For surveys conducted between 2015 and 2017, studies directly conducted by the Korean government for public welfare were exempt from review by the KDCA IRB.

### 2.2. Demographic Information

Demographic and lifestyle information was obtained from the health questionnaire survey [14]. The household income level was divided into low, mid-low, mid-high, and high quartiles according to the average monthly equalized household income. The obtained information on education level was categorized into elementary school or lower, middle school graduation, high school graduation, and college graduation or higher. We recategorized this information into middle school graduation or lower and high school graduation or higher. Based on the current smoking status, individuals were classified as smokers and non-smokers. Alcohol consumption was determined as the number of servings of alcohol consumed per day calculated by multiplying the number of servings per sitting and frequency of alcohol consumption in the past year. Alcohol drinkers were defined as individuals who drank >0 g of alcohol. Regarding the DM medical treatment status, individuals were classified as those who took DM medication or injected insulin and those who did not. To calculate physical activity level, metabolic equivalent of tasks (METs-h/week) was calculated based on the number of days and hours of intense, moderate, and walking physical activity by assigning a weighted value [15]. The DM duration was calculated by subtracting the age at DM diagnosis from the current age.

### 2.3. Dietary Intake and SSC Consumption

Nutrient intake levels, including the total energy, carbohydrate, protein, and fat, were estimated based on the 24 h recall data [14]. The 24 h recall method of the KNHANES was used by trained interviewers who visited participating households during the examination [14]. The collected food and beverage intake information was converted into individual food items using the food recipe database published by the Korea Health Industry Development Institute [2]. In total, 1304 food codes were classified as “beverages and teas,” of which 123 food codes containing “coffee” were selected. SSC consumption was defined as drinking any type of coffee with added sugar, syrup, or honey, such as ground coffee or instant powder coffee with sugar, syrup, or honey; 3-in-1 coffee; SSC drinks; or canned coffee. Total energy, carbohydrate, protein, and fat intakes were compared with the 2020 Dietary Reference Intakes for Koreans (KDRIs) [16]. The percentage of estimated total energy requirement and proportion of energy from carbohydrates, proteins, and fats were calculated.

### 2.4. Anthropometric Measurements and Health Indicators

Body mass index (BMI) was calculated as body weight (kg) divided by height squared (m^2^) [15]. Venous blood was collected after 8 h of fasting [17]. FBG levels were analyzed using the hexokinase method. The blood analytic instruments used in the KNHANES were ADIVIA 1650 (Siemens, Tarrytown, NY, USA) in 2008–2009, Hitachi Automatic Analyzer 7600 (Hitachi, Tokyo, Japan) in 2010–2012, Hitachi Automatic Analyzer 7600-210 (Hitachi, Tokyo, Japan) in 2013–2018, and Labospect 008AS (Hitachi, Tokyo, Japan) in and after 2019 [14]. The clinical test for HbA1c level was performed by high-performance liquid chromatography using HLC-723G7 (Tosoh, Tokyo, Japan) in 2008–2012 and Tosoh G8 (Tosoh, Tokyo, Japan) in and after 2013 along with appropriate reagents.

### 2.5. Definition of Glycemic Target Achievement for DM

Achievement of glycemic target for DM was defined as an FBG level of 80–130 mg/dL or HbA1c level of <6.5% based on the “glycemic targets for adults” set by the “Clinical Practice Guidelines for Diabetes” and “Treatment Guidelines for Diabetes” issued by the Korean Diabetes Association [18,19,20].

### 2.6. Statistical Analysis

All statistical analyses were performed considering KNHANES’s complex survey design, sampling weights, and stratified and clustered sampling approaches [14]. Categorical variables are shown as frequencies and percentages, and the chi-square test was used for significance testing. Continuous variables are shown as mean ± standard error, and linear regression analysis was used for significance testing. Multivariate linear regression analysis was performed to estimate the mean FBG and HbA1c levels according to SSC consumption. Potential confounding factors were identified using preliminary analysis and literature review [21,22,23,24,25,26,27]. Multiplicative terms were used in the statistical model to identify the effect modifiers; however, no effect modifier of the association between SSC consumption and the glycemic target was found.

To analyze the association between SSC consumption and achievement of the glycemic target, multivariate logistic regression analysis was performed to calculate the odds ratio (OR) and 95% confidence interval (CI). Consequently, the following models were built to consider potential confounding factors: Model 1, unadjusted; Model 2, adjusted for sex and age; and Model 3, adjusted additionally for smoking and drinking status, household income, education level, physical activity level, DM medical treatment status, BMI, DM duration, and total energy intake. *p* for trend was computed by median values for SSC consumption. All statistical analyses were performed using the Statistical Analysis System version 9.4 (SAS Institute, Cary, NC, USA). Statistical significance was set at α = 0.05.

## 3. Results

### 3.1. General Characteristics of the Participants According to the Frequency of SSC Consumption

The number of individuals who consumed SSC zero, one, and two or more times/day was 3030, 1501, and 1140, respectively (Table 1). Among all people with DM included in the analysis, 46.57% drank SSC. In the two or more times/day SSC consumption group, 69.82% individuals were men, a proportion higher than that in the other groups. The proportion of study participants currently consuming alcohol was approximately 50%, with 51.05%, 56.09%, and 63.65% individuals in the zero, one, and two or more times/day SSC consumption groups, respectively. The proportion of study participants who currently smoked was different among the groups, with higher current smokers in the one time/day (16.01%) and two or more times/day (33.87%) SSC consumption groups than in the zero time/day SSC consumption group (11.68%) (*p* < 0.001). Patients with DM in the two or more times/day SSC consumption group tended to have higher total energy intakes (1941.49 ± 20.75) than those in the one time/day (1699.79 ± 18.08) and zero time/day (1667.31 ± 12.73) SSC consumption groups.

### 3.2. Total Energy and Macronutrients Intake According to the Frequency of SSC Consumption

Table 2 shows the percentage of estimated total energy requirement and proportion of energy from carbohydrates, proteins, and fats according to the frequency of SSC consumption. The percentage of estimated total energy requirement was higher in the two or more times/day SSC consumption group than in the other groups (*p* value < 0.001). There were no significant differences in the proportion of energy from carbohydrates and fats between the SSC consumption groups; however, the proportion of energy from proteins was lower in the two or more times/day SSC consumption group than in the other groups (*p* value < 0.001).

### 3.3. Mean FBG and HbA1c Levels According to the Frequency of SSC Consumption

**Figure 1:**
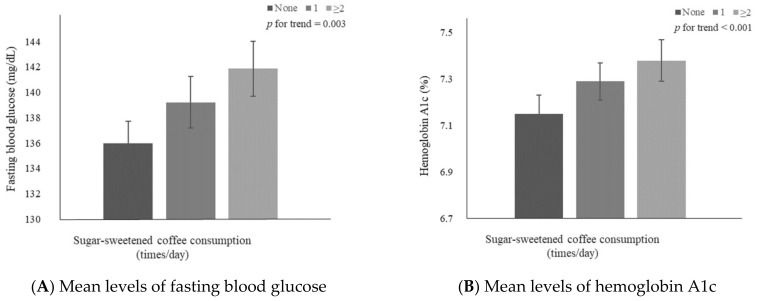
Mean levels of (**A**) fasting blood glucose and (**B**) hemoglobin A1c according to the frequency of sugar-sweetened coffee consumption. Values were adjusted for age (continuous), sex, education level (middle school graduation or lower and high school graduation or higher), household income (low, mid-low, mid-high, and high), smoking status (non-smokers and smokers), alcohol consumption (non-drinkers and drinkers), physical activity (continuous), DM medical treatment status (oral hypoglycemic agents or insulin treatments and no treatment), body mass index (continuous), DM duration (continuous), and total energy intake (continuous).

Figure 1 shows multivariable-adjusted means of FBG and HbA1c levels in individuals with DM according to the frequency of SSC consumption. The results showed significant increase patterns in mean FBG (*p* for trend = 0.003) and HbA1c (*p* for trend < 0.001) levels with an increase in the frequency of SSC consumption. The adjusted means ± standard errors were 135.96 ± 1.75, 139.21 ± 2.04, and 141.87 ± 2.17 for FBG level and 7.15 ± 0.08, 7.29 ± 0.08, and 7.38 ± 0.09 for HbA1c level in the zero, one, two or more times/day SSC consumption groups, respectively.

### 3.4. Glycemic Target Levels Achieved by Participants with DM

Table 3 shows the association between SSC consumption and achievement of the glycemic targets by participants with DM. The proportion of individuals who did not achieve the FBG target was 48.22%, 50.62%, and 52.03% in the zero, one, two or more times/day SSC consumption groups, respectively. After adjusting for all covariates, the OR of not achieving the FBG target was significantly higher in the two or more times/day (OR: 1.24, 95% CI: 1.03–1.48) and one time/day (OR: 1.18, 95% CI: 1.01–1.38) SCC consumption groups than in the zero time/day SSC consumption group (*p* for trend = 0.01) group.

The proportion of individuals who did not achieve the HbA1c target was 71.19%, 74.08%, and 74.82% in the zero, one, two or more times/day SSC consumption groups, respectively. After adjusting for all covariates, the OR of not achieving the HbA1c target was significantly higher in the two or more times/day SSC consumption group (OR: 1.29, 95% CI: 1.05–1.58) than in the zero time/day SSC consumption group (*p* for trend = 0.009), but no significant difference was observed between zero and one time/day SSC consumption groups (OR: 1.15, 95% CI: 0.96–1.38).

## 4. Discussion

Among Korean participants with DM who participated in the KNHANES (2008–2020) for the past 13 years, approximately 46.57% consumed SSC. Participants who consumed SSC tended to have higher FBG and HbA1c levels and a higher OR of not achieving the glycemic targets required for DM management than those who did not consume SSC. 

We found that participants with frequent SSC intake tended to have higher total energy and lower protein intake levels. We assume that this finding can be explained by the increased added sugar consumption from SSC. Patients with DM need to control their total calorie intake to maintain a normal weight. In addition, it is important to consume unrefined, high-quality carbohydrates along with appropriate portions of protein and fat and avoid sugar-sweetened food products [18,19]. Consumption of sufficient good quality protein is strongly recommended for patients with DM to prevent various health problems such as decline in skeletal muscle mass or diabetic complications [28,29,30,31]. A pooled analysis of two Japanese cohort studies (the Japan Diabetes Complications Study and Japanese Elderly Diabetes Intervention Trial) has shown that low protein intake is associated with a higher all-cause mortality, particularly among older adults aged ≥75 years [32].

Cumulative evidence has consistently shown that coffee consumption can reduce not only the occurrence of chronic diseases but also various risks of death. In a pooled analysis of 12 prospective cohort studies including 477,192 Asians, all-cause mortality was lower by 24% among men and 28% among women who consumed five or more cups of coffee a day than among those who consumed less than one cup of coffee a day [33]. Similarly, a meta-analysis of 10 cohort studies including patients with DM reported an inverse association between the frequency of coffee consumption and all-cause mortality [34]. In contrast, several cross-sectional studies conducted in Korea have reported that coffee consumption is associated with a higher prevalence of obesity, abdominal obesity, and metabolic syndrome [35,36,37]. In a study conducted by Korean National Cancer Center including 5995 women aged 30–70 years, women who consumed three or more cups of coffee per day had lower prevalent obesity and abdominal obesity than those who did not consume coffee, emphasizing that such association might be attributed to the type of coffee and coffee additives consumed [35]. Furthermore, a cross-sectional study analyzing KNHANES data reported that the prevalence of obesity and abdominal obesity was higher among women who consumed three or more cups of coffee a day than among those who consumed less than 1 cup per day [36]. Moreover, a study by Kim et al., which included 42,347 participants from the KNHANES, showed a higher prevalence of metabolic syndrome among people who consumed instant coffee with sugar and creamer than among those who did not (OR: 1.21, 95% CI: 1.02–1.43) [37].

The scientific communities have focused on the benefits of diabetes-related risk of polyphenols in coffee [38]. Polyphenols inhibit glucose release from the liver and reduce secretion of gastric inhibitory polypeptide to regulate FBG and plasma glucose peak by decreasing glucose uptake in the intestines [39]. However, some studies suggested that caffeine intake may decrease insulin sensitivity in patients with DM [40]. A meta-analysis of seven randomized controlled studies showed that, in the short term, caffeine intake reduced insulin sensitivity [41]. Caffeine is known to reduce insulin sensitivity by the antagonism of adenosine receptors in skeletal muscles in adults, and an increase in plasma epinephrine levels in response to caffeine intake also reduces insulin sensitivity [42,43]. Caffeine consumption also appears to have diverse health effects. As previous studies have shown, consuming high levels of caffeine may increase the heart rate [44,45] and energy expenditure [46], showing that fat oxidation is more likely to occur in individuals with higher caffeine consumption, resulting in weight loss [47].

One of the health concerns of frequent SSC intake is that sugar and high-fructose corn syrup (HFCS), an additive in beverages, can have negative health effects. Consuming excessive amount of fructose over a short period can lower the hepatic insulin sensitivity index [48], which can increase the blood insulin level. A ecological perspective study of 43 countries including Korea, showed that the prevalence of DM was high in countries with higher HFCS consumption [49]. Moreover, in a double-blinded randomized controlled study, consumption of fructose-sweetened beverages increased hepatic lipogenesis and visceral fat, unlike consumption of glucose-sweetened beverages [50]. Drinking coffee has been connected to the positive effect on blood glucose level; however, adding sugar to coffee weakens this beneficial effect and may induce glycemic dysregulation. In addition, individuals with DM have a higher risk of coronary artery disease than those without DM [51]. In a meta-analysis of 102 prospective studies, individuals with DM had a higher risk of coronary heart disease and stroke than those without DM. In another meta-analysis of 77 prospective studies, there was a significant positive association between DM diagnosis and risk of heart failure (relative risk: 2.06, 95% CI: 1.73–2.46) [52]. This could be attributed to impaired glucose tolerance in patients with DM, causing vascular endothelial dysfunction and decreased vascular compliance, which could increase the pulse wave velocity of central arteries to show a negative correlation with plasma adiponectin levels [53]. Moreover, DM-induced vascular problems can also cause circulatory impairment and kidney, eye, and neurological disorders [54]. In particular, patients with DM have a higher tendency for obesity, which also causes or exacerbates diabetic complications due to concomitant abnormalities such as high insulin resistance, than those without DM [55].

Improper management of blood glucose level can also cause various complications in patients with DM. A cohort study of 3099 Korean outpatients with DM at the Veteran Health Service Medical Center from 2008 to 2017 showed an increased risk of chronic kidney disease and end-stage renal disease among outpatients with ≥8.5% HbA1c levels than those <6.5% HbA1c levels [56]. In addition, other studies have reported that improper glucose level management or prolonged disease duration due to poor management may be associated with cardiovascular diseases, sexual dysfunction, reduced total gray matter volume, dementia, and gastrointestinal diseases such as dyspepsia [51,57,58,59,60,61,62]. Therefore, it is necessary to control SSC intake among patients with DM for glycemic control and prevention of complications.

To the best our knowledge, this is the first study in Korea on SSC consumption and glycemic control among Korean patients with DM. The use of data from the KNHANES, a South Korean nationally representative cross-sectional survey, enhanced the generalizability of our results. However, this study has some limitations. First, the association between SSC consumption and the glycemic target in patients with DM was analyzed using cross-sectional data; therefore, the cause–effect relationship could not be determined. However, as the study population included patients with DM, the likelihood of reverse causality of SSC consumption based on presence or absence of disease is low. Second, SSC consumption was estimated by using 1-day 24 h recall, which might not have accurately reflected the participants’ usual intake levels. Third, although the confounding factors identified through literature review and preliminary data analysis, there might be possibilities of residual confounding that might have influenced the association between SSC consumption and achievement of the glycemic target. Lastly, potential measurement errors might exist due to differences in the instruments used for blood analysis in different survey years. However, trained investigators collected data and assessed its quality to increase the reliability and validity of the KNHANES [63].

## 5. Conclusions

In conclusion, approximately 50% of Korean patients with DM consume SSC, which may cause difficulties in blood glucose control. Our study shows that patients with DM with frequent SSC consumption have higher FBG and HbA1c levels and are less likely to achieve the glycemic targets needed for DM management than those consumed SSC less frequently. Our results emphasize the need for professional education on glycemic control and dietary habits for such patients. Moreover, the study findings are expected to be used as basic data for managing dietary habits of patients with DM and as reference material for future studies.

## Figures and Tables

**Table 1:** Demographic and lifestyle characteristics of participants with diabetes mellitus according to the frequency of sugar-sweetened coffee consumption, KNHANES 2008–2020.

	Frequency of SSC Consumption (Day)			*p*-Value
Characteristics	None	1	≥2	
N (%)	3030 (53.43)	1501 (26.47)	1140 (20.10)	
Age	64.99 ± 0.19	66.28 ± 0.27	64.51 ± 0.31	0.9
Sex				<0.001
Men	1270 (41.91)	671 (44.70)	796 (69.82)	
Women	1760 (58.09)	830 (55.30)	344 (30.18)	
Household income				0.003
Low	1121 (37.29)	584 (39.25)	396 (34.92)	
Mid-low	811 (26.98)	415 (27.89)	299 (26.37)	
Mid-high	536 (17.83)	275 (18.48)	252 (22.22)	
High	538 (17.90)	214 (14.38)	187 (16.49)	
Education level				0.04
Middle school graduation or lower	1821 (62.28)	952 (65.52)	675 (61.14)	
High school graduation or higher	1103 (37.72)	501 (34.48)	429 (38.86)	
Alcohol consumption status				<0.001
Non-drinkers	1463 (48.95)	653 (43.91)	410 (36.35)	
Drinkers	1526 (51.05)	834 (56.09)	718 (63.65)	
Smoking status				<0.001
Non-smokers	2640 (88.32)	1249 (83.99)	744 (66.13)	
Smokers	349 (11.68)	238 (16.01)	381 (33.87)	
DM medical treatment status				0.09
Oral hypoglycemic agents or insulin treatments	2757 (90.99)	1379 (91.87)	1019 (89.39)	
Non-care	273 (9.01)	122 (8.13)	121 (10.61)	
Physical activity level (METs-h/week)	24.64 ± 0.80	25.24 ± 1.13	27.12 ± 1.30	0.1
Body mass index (kg/m^2^)	24.96 ± 0.06	24.99 ± 0.09	24.96 ± 0.10	0.9
Diabetes duration (year)	9.35 ± 0.16	9.51 ± 0.22	9.36 ± 0.25	0.8
Total energy intake (kcal/day)	1667.31 ± 12.73	1699.79 ± 18.08	1941.49 ± 20.75	<0.001

KNHANES, Korea National Health and Nutrition Examination Survey. SSC, Sugar-Sweetened Coffee. Values are presented as N (%) or mean ± standard error. *p*-Values were derived from the chi-square test for categorical variables, and *p* for trend was derived from generalized linear regression analysis for continuous variables.

**Table 2:** Total energy and macronutrient intake values according to the frequency of sugar-sweetened coffee consumption.

Frequency of SSC Consumption (Day)	None	1	≥2	*p*-Value
Total energy (%, KDRIs) ^(1)^	91.94 ± 1.37 ^B^	95.07 ± 1.59 ^B^	99.69 ± 1.65 ^A^	<0.001
Carbohydrate (% of total energy) ^(2)^	69.76 ± 0.42	69.97 ± 0.47	70.54 ± 0.46	0.2
Protein (% of total energy) ^(2)^	14.63 ± 0.20 ^A^	14.12 ± 0.20 ^B^	13.57 ± 0.21 ^C^	<0.001
Fat (% of total energy) ^(2)^	15.62 ± 0.32	15.92 ± 0.37	15.89 ± 0.34	0.6

KDRIs, Dietary Reference Intakes for Koreans. SSC, Sugar-Sweetened Coffee. Values are presented as mean ± standard error. ^(1)^ Values were adjusted for age (continuous), sex, education level (middle school graduation or lower and high school graduation or higher), household income (low, mid-low, mid-high, and high), smoking status (non-smokers and smokers), alcohol consumption (non-drinkers and drinkers), DM medical treatment status (oral hypoglycemic agents or insulin treatments and no treatment), physical activity (continuous), body mass index (continuous), and DM duration (continuous). ^(2) (1)^ plus additional adjustment for total energy intake (continuous). Different letters indicate significant differences with the Tukey–Kramer multiple comparison test (*p* < 0.05).

**Table 3:** Odds ratios (95% confidence intervals) for not achieving the glycemic targets by patients with type 2 diabetes mellitus according to the frequency of sugar-sweetened coffee consumption, KNHANES 2008–2020.

Frequency of SSC Consumption (Day)	None	1	≥2	*p* for Trend
**Fasting blood glucose**				
Cases (%)	1421 (48.22)	736 (50.62)	565 (52.03)	
Model 1	Ref	1.18 (1.01–1.36)	1.25 (1.06–1.48)	0.005
Model 2	Ref	1.21 (1.04–1.40)	1.20 (1.01–1.43)	0.01
Model 3	Ref	1.18 (1.01–1.38)	1.24 (1.03–1.48)	0.01
**Hemoglobin A1c**				
Cases (%)	2157 (71.19)	1112 (74.08)	853 (74.82)	
Model 1	Ref	1.16 (0.98–1.37)	1.26 (1.04–1.52)	0.009
Model 2	Ref	1.18 (1.00–1.40)	1.28 (1.06–1.56)	0.005
Model 3	Ref	1.15 (0.96–1.38)	1.29 (1.05–1.58)	0.009

SSC, Sugar-Sweetened Coffee. Glycemic targets, fasting blood glucose level 80–130 mg/dL or hemoglobin A1c level <6.5%. Model 1, Unadjusted; Model 2, Adjusted for sex and age (continuous); Model 3, Model 2 plus additional adjustment for education level (middle school graduation or lower and high school graduation or higher), household income (low, mid-low, mid-high, and high), smoking status (non-smokers and smokers), alcohol consumption (non-drinkers and drinkers), DM medical treatment status (oral hypoglycemic agents or insulin treatments and no treatment), physical activity (continuous), body mass index (continuous), DM duration (continuous), and total energy intake (continuous).

## Data Availability

The datasets supporting the conclusions of this article are available from the Korea Centers for Disease Control and Prevention on reasonable request. These datasets are available with a permission at the following URLs: https://knhanes.kdca.go.kr/knhanes/sub03/sub03_02_05.do (accessed on 12 November 2022).
